# Reduced cGMP levels in CSF of AD patients correlate with severity of dementia and current depression

**DOI:** 10.1186/s13195-017-0245-y

**Published:** 2017-03-09

**Authors:** Raphael Hesse, Ludwig Lausser, Pauline Gummert, Florian Schmid, Anke Wahler, Cathrin Schnack, Katja S. Kroker, Markus Otto, Hayrettin Tumani, Hans A. Kestler, Holger Rosenbrock, Christine A. F. von Arnim

**Affiliations:** 1 Department of Neurology, Ulm University, Oberer Eselsberg 45, 89081 Ulm, Germany; 2 Department of CNS Diseases Research, Boehringer Ingelheim Pharma GmbH & Co KG, Biberach, Germany; 3 Department of Drug Discovery Support, Boehringer Ingelheim Pharma GmbH & Co KG, Biberach, Germany; 4 Institute of Medical Systems Biology, Ulm University, Ulm, Germany

**Keywords:** Alzheimer’s disease, Cerebrospinal fluid, cognitive decline, cyclic nucleotides, Neuropsychiatric symptoms

## Abstract

**Background:**

Alzheimer’s disease (AD) is a neurodegenerative disorder, primarily affecting memory. That disorder is thought to be a consequence of neuronal network disturbances and synapse loss. Decline in cognitive function is associated with a high burden of neuropsychiatric symptoms (NPSs) such as depression. The cyclic nucleotides cyclic adenosine-3',5'-monophosphate (cAMP) and cyclic guanosine-3',5'-monophosphate (cGMP) are essential second messengers that play a crucial role in memory processing as well as synaptic plasticity and are potential therapeutic targets. Biomarkers that are able to monitor potential treatment effects and that reflect the underlying pathology are of crucial interest.

**Methods:**

In this study, we measured cGMP and cAMP in cerebrospinal fluid (CSF) in a cohort of 133 subjects including 68 AD patients and 65 control subjects. To address the association with disease progression we correlated cognitive status with cyclic nucleotide levels. Because a high burden of NPSs is associated with decrease in cognitive function, we performed an exhaustive evaluation of AD-relevant marker combinations in a depressive subgroup.

**Results:**

We show that cGMP, but not cAMP, levels in the CSF of AD patients are significantly reduced compared with the control group. Reduced cGMP levels in AD patients correlate with memory impairment based on Mini-Mental State Examination score (*r* = 0.17, *p* = 0.048) and tau as a marker of neurodegeneration (*r* = –0.28, *p* = 0.001). Moreover, we were able to show that AD patients suffering from current depression show reduced cGMP levels (*p* = 0.07) and exhibit a higher degree of cognitive impairment than non-depressed AD patients.

**Conclusion:**

These results provide further evidence for an involvement of cGMP in AD pathogenesis and accompanying co-morbidities, and may contribute to elucidating synaptic plasticity alterations during disease progression.

**Electronic supplementary material:**

The online version of this article (doi:10.1186/s13195-017-0245-y) contains supplementary material, which is available to authorized users.

## Background

The cyclic nucleotides cyclic adenosine-3′,5′-monophosphate (cAMP) and cyclic guanosine-3′,5′-monophosphate (cGMP) are essential second messengers and play a crucial role in memory processing and synaptic plasticity. Both cAMP and cGMP are important elements in intracellular signal transduction cascades, mediating effects mainly by activating protein kinases (PKA and PKG) and ion channels [1]. Activation of these cascades leads to phosphorylation of the synaptic plasticity-related transcription factor cAMP-response element binding protein (CREB) [2]. CREB activation by phosphorylation at Ser133 has been shown to be necessary for strengthening synaptic plasticity and long-term memory formation [3]. Intracellular cAMP/cGMP levels are tightly regulated by synthesis from adenosine 5′-triphosphate/guanosine 5′-triphosphate via adenylyl/guanylyl cyclases and degradation via phosphodiesterases (PDEs), which catalyses the hydrolysis of the cyclic nucleotides to the corresponding 5′-monophosphates [1]. Alzheimer’s disease (AD) is a neurodegenerative disorder, affecting memory encoding and storage-related brain regions such as the hippocampus and cortical areas [4]. AD is histochemically defined by Aβ plaques and neurofibrillary tangles consisting of tau [5]. Soluble Aβ aggregates seem to affect hippocampal synaptic plasticity, and affect synapse and memory loss via CREB signalling [6]. Since the early 1990s, the interest in cGMP modulating PDEs has increased remarkably, as the number of PDE inhibitors in clinical trials for the treatment of several vascular as well as neurological disorders has grown considerably [7]. In fact, mice treated with PDE9 inhibitors (cGMP specific) exhibit improved learning and memory [8], and inhibition of PDE9A further leads to a rescue of Aβ-induced deficits in synaptic plasticity and cognition in the APP transgenic mouse model tg2576 [9]. Changes in cyclic nucleotide signalling due to altered PDE expression in AD patients may therefore lead to an altered level of CREB-induced neuroprotection [10].

Given that AD is characterized by a progressive loss of synapses leading to dementia, cyclic nucleotides could serve as promising biomarkers because synaptic function is closely modulated by cGMP signalling, which seems to be reduced in the brains of AD patients [11]. Thus, selective inhibition of PDEs could offer novel approaches in the therapy of people suffering from dementia. To monitor therapy and to determine disease development and progression, one focus in biomedical research of neurodegenerative diseases is the identification of novel biomarkers [12, 13]. Further, because the CSF compartment is close to the neuropathological changes due to AD, this makes it the preferred body fluid for identifying novel biomarkers for brain-related diseases. A recent study reported that the decreased levels of cGMP in the CSF of AD patients are associated with cognitive decline and amyloid pathology [14]. However, another study reported an increase in cAMP, but not in cGMP, levels in the CSF of AD patients [15]. Both studies were limited in terms of cohort size (*n* = 79 and *n* = 20 respectively).

Although cognitive function is primarily known to be impaired in AD patients, there is evidence that extensive neuronal connections exist between the neural regions of cognition/memory (hippocampus) and emotions (amygdala) [16, 17]. This relationship is in line with a recent study showing a high prevalence of late-life depressive symptoms in dementia [18]. In addition, there is strong evidence that the glutamatergic system, including cGMP signalling, is involved in the neuropathology of depressive symptoms in AD [19, 20].

To further elucidate the role of cyclic nucleotides in AD and accompanying neuropsychiatric symptoms (NPSs) such as cognitive decline and depression, we measured the cGMP and cAMP levels in the CSF in a large cohort comprised of 68 AD patients and 65 non-demented control subjects. We evaluated whether cAMP/cGMP levels correlate with disease parameters such as cognitive function based on Mini-Mental State Examination (MMSE) score and current depression based on the Beck Depression Inventory (BDI). The diagnostic power of these parameters was also determined. This article confirms the involvement of cGMP in AD progression in a highly selective cohort and reveals—to the best of our knowledge for the first time—a connection between CSF cGMP and late-life depressive symptoms in AD patients.

## Methods

### Participants

Patient cohorts were recruited between 2003 and 2015 at the Memory Clinic of the Neurology University Hospital in Ulm, Germany. CSF was collected by lumbar puncture at the same institute. All participants underwent a comprehensive clinical neurological examination and a detailed neuropsychological assessment including the MMSE as a general screening test [21] and the BDI and Geriatric Depression Scale (GDS) as tests for current depression. ApoE ε allele status was also determined. Exclusion criteria were a history of stroke or any other reason for cognitive impairment, other neurodegenerative diseases, a history of alcohol or drug abuse, visual or linguistic impairment and cephalalgia. The diagnostic criteria were defined according to the NIA–AA criteria [22–24], which use a combination of clinical diagnosis and the CSF biomarker profile including Aβ_1–42_ and T‐tau. Only patients fulfilling the diagnostic criteria for “probable” AD were included. Inclusion criteria were CSF tau > 350 pmol/ml, CSF Aβ < 650 pmol/ml and MMSE score ≤ 25. Unrelated control subjects were recruited at the same site.

#### Control subjects

The group of controls comprised 65 cognitively normal individuals (37 male and 28 female) who presented at the memory clinic with normal CSF core biomarkers (CSF tau < 350 pmol/ml, CSF Aβ > 650 pmol/ml, MMSE score ≥ 25) with a median (IQR) age of 59 (54–71) years. The control group showed no evidence of stroke, (history of) headache or neuroinflammatory or other neurodegenerative diseases according to the evaluation of a neurologist.

The diagnosis of current depression was made by applying BDI (cut-off value ≥ 13) or GDS (cut-off value ≥ 5). The second inclusion criterion was administration of antidepressants. Twenty-five of the AD patients suffered from depression.

Demographic details of all patients are presented in Table 1.

**Table 1:** Patient demographic data

	Control (*n* = 65)	AD (*n* = 68)	*p* value
Age (years)	59 (54–71)	70 (66–75)	<0.01
Sex, male/female (*n*)	37/28	25/43	0.021
ApoE ε4 carriers (%)	23.1	52.9	
Tau (pg/ml)	229.5 (172.25–292)	907 (641.5–1100.5)	<0.01
Aβ_42_ (pg/ml)	1012.5 (850.75–1177.25)	493 (390.5–556)	<0.01
MMSE score	29 (27–30)	20 (16–23)	<0.01

Data presented as median (interquartile range) unless otherwise stated. *p* values calculated using the Mann–Whitney test, AD vs control

*AD* Alzheimer’s disease, *MMSE* Mini-Mental State Examination

### Standard protocol approvals, registrations and patient consent

The study was approved by the university ethics board (No. 2001/67). All individuals gave written informed consent to their participation in the study.

### Measurement of CSF β-amyloid(42) and tau levels

CSF collection and pre-analytic processing were performed using a standardized protocol as described previously [25]. Briefly, CSF samples were collected in propylene tubes after lumbar puncture and centrifuged immediately after collection. Samples were stored at –80 °C in Eppendorf tubes within 2 h until further analysis. Standard sandwich ELISA techniques were used to quantify the CSF levels of β-amyloid(42) (Innotest β-amyloid 1–42) [26] and t-tau (Innotest hTau-Ag) [27] following the manufacturer’s instructions.

### Enzyme-linked immunosorbent assay

CSF levels of cAMP and cGMP were determined by ELISA. Both cAMP and cGMP ELISAs were obtained from Enzo Lifesciences (cAMP: #ADI-900-066; cGMP: #ADI-900-014) and used according to the manufacturer’s protocol. Briefly, for cAMP, 100 μl standard or CSF and 100 μl 0.1 M HCl were added into the appropriate wells. For cGMP, 200 μl CSF and 200 μl 0.1 M HCl were used. After adding 50 μl of the conjugate and 50 μl of the antibody to the appropriate wells, the plates were incubated at room temperature for 2 h with shaking. After washing steps, pNpp substrate and stop solution were added and the optical density was measured at 405 nm. The assays were blind for patient identification and disease status.

### Statistics

Statistical analyses were carried out with GraphPad Prism version 6.05. Compared groups were tested for normality (Kolmogorov–Smirnov test) and failed. Groups were compared by Mann–Whitney *U* rank-sum test or Kruskal–Wallis test when more than two groups were compared. Correlation of parameters was calculated using Spearman’s rank correlation coefficient. *p* < 0.05 was considered statistically significant. The results are expressed as median (25th–75th percentile).

Classification experiments were performed using R Version 3.3.0 [28] and the TunePareto R package [29]. We analysed the predictive performance of combinations of markers in an AD vs control scenario on a subgroup of patients with depression. The utilized classes consisted of 19 AD samples and 33 control samples, all with depression. Seven parameters were used for the experiments: total protein, albumin, ApoE4 status, cGMP, cAMP, age and thyroid problems. The predictive performance of all combinations (*n* = 2^7^ – 1 = 127) was determined in a cross-validation experiment (10 × 10 folds) for each parameter combination. In this experiment the data set is split into 10 folds of approximately equal size. Nine folds are used for training a model, and the remaining fold is used for testing the model. This is repeated until each fold has been used once as test fold. To exclude any influence of the sample order, 10 repetitions have been performed using random permutations of the samples. A classification tree (CART) based on the Gini index was utilized as a base classifier [30]. As a performance measure, the mean of sensitivity and specificity was used because classes are not balanced.

## Results

### CSF levels of cGMP, but not cAMP, are decreased in AD patients

**Fig. 1:**
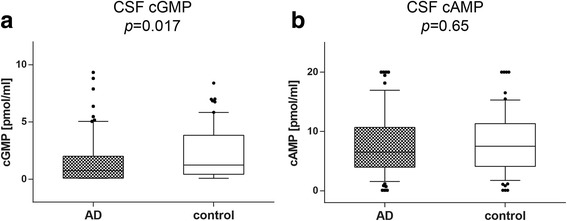
cGMP, but not cAMP, levels in CSF of AD patients are reduced. Box plots comparing CSF levels of cGMP (**a**) and cAMP (**b**) in CSF of patients with AD compared with control patients measured by ELISA. (**a**) cGMP levels in the CSF of AD patients were significantly reduced compared with control subjects (*p* = 0.017). (**b**) cAMP levels in the CSF of AD patients were not altered. Mann–Whitney *U* test. *AD* Alzheimer’s disease, *cAMP* cyclic adenosine-3',5'-monophosphate, *cGMP* cyclic guanosine-3',5'-monophosphate, *CSF* cerebrospinal fluid

The ELISA analysis of cGMP and cAMP levels in the CSF of AD patients revealed a significant reduction of about 40% in cGMP levels (*p* = 0.017; Fig. 1a). The median concentration was 1.23 (0.43–3.84) pmol/ml (control) vs 0.75 (0.09–2.01) pmol/ml (AD). cAMP levels were not altered in the CSF of AD patients compared with the controls (Fig. 1b) (7.51 pmol/ml vs 6.51 pmol/ml, n.s.) (AD).

### Reduction in cGMP levels in CSF of AD patients correlates with lower MMSE score and negatively correlates with CSF tau

**Fig. 2:**
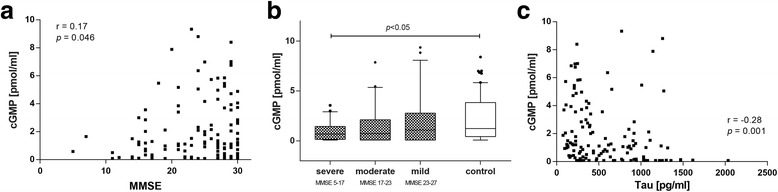
Only severely demented AD patients show a significant decrease in CSF levels of cGMP. **a** Correlation analysis of CSF cGMP levels with the cognitive performance (MMSE score) of all participants showed a significant correlation of CSF cGMP levels and MMSE scores (*p* = 0.046, Spearman’s rank correlation coefficient). **b** CSF levels of cGMP of control subjects and AD patients stratified by their MMSE scores (severe AD = MMSE 5–17, moderate AD = MMSE 17–23, mild AD = MMSE 23–27). Only severely demented AD patients show a significant reduction in CSF cGMP levels compared with the control group (*p* < 0.01, Mann–Whitney *U* test). **c** Correlation of CSF cGMP levels with neurodegeneration marker tau showed a significantly negative correlation (*p* = 0.001, Spearman’s rank correlation coefficient). *cGMP* cyclic guanosine-3',5'-monophosphate, *MMSE* mini-mental state examination

Because cGMP is closely related to memory function we next examined the correlation between CSF cGMP levels and the cognitive performance of all participants (MMSE score). A statistically significant correlation was observed (*r* = 0.17, *p* = 0.046; Table 2, Fig. 2a). To obtain more detailed information about the relationship between cGMP levels and severity of dementia, AD patients were stratified by their MMSE scores into three groups (severe AD, moderate AD, mild AD). The first tertile comprises AD patients with an MMSE score of 5–17 (severe AD; *n* = 23), the second tertile patients with an MMSE score of 17–23 (moderate AD; *n* = 23) and the third tertile comprises AD patients with an MMSE score of 23–27 (mild AD; *n* = 22). We observed a trend of reduction in mild dementia (~10%) and in moderate dementia (~40%). The comparison of severe dementia with the lowest MMSE scores showed a significant decrease in cGMP levels of about 50% compared with the controls (*p* < 0.05; Fig. 2b). The median concentration was 1.23 (0.43–3.84) pmol/ml (control), 1.08 (0.08–2.78) pmol/ml (severe AD), 0.72 (0.08–2.12) pmol/ml (moderate AD) and 0.69 (0.16–1.45) pmol/ml (mild AD). Furthermore, the decrease in CSF cGMP negatively correlates significantly with CSF tau levels (*r* = 0.28, *p* = 0.001; Table 2, Fig. 2c).

**Table 2:** Correlations between CSF cyclic nucleotide levels and clinical and pathological markers of AD

	CSF cGMP		CSF cAMP	
	*r*_s_	*p*	*r*_s_	*p*
CSF Aβ42	0.15	0.09	0.056	0.5183
CSF tau	**–0.28**	**0.001**	–0.067	0.438
MMSE score	**0.17**	**0.046**	–0.047	0.59

Correlation coefficients (*r*_s_) and *p* values calculated using Spearman’s rank correlation analysis. Statistically significant results depicted in bold

*AD* Alzheimer’s disease, *cAMP* cyclic adenosine-3',5'-monophosphate, *cGMP* cyclic guanosine-3',5'-monophosphate, *CSF* cerebrospinal fluid, *MMSE* mini-mental state examination

### Capability of cGMP in CSF as a biomarker of AD

**Fig. 3:**
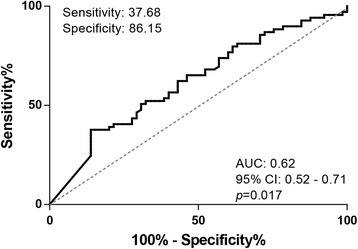
Diagnostic potential of cGMP as an AD biomarker. ROC curve analysis of CSF levels of cGMP in AD patients compared with control subjects. Sensitivity is defined as the fraction of those with the disease correctly identified as positive by the test. Specificity is defined as the fraction of those without the disease correctly identified as negative by the test. Youden’s index = sensitivity + specificity – 1. Likelihood ratio = sensitivity / (1 – specificity). Cut-off value for cGMP was <0.201 pmol/ml. *AUC* area under the curve, *CI* 95% confidence interval

To test the capability of cGMP as a potential biomarker of AD we calculated the receiver operating characteristic (ROC) curve. The ROC curve is depicted in Fig. 3. According to ROC curve analysis, the CSF levels of cGMP discriminate the AD patients from control subjects with a specificity of 86.15% and a sensitivity of 37.68% (AUC = 0.62, 95% CI: 0.52–0.71, *p* = 0.017). The cut-off value was calculated using Youden’s index and the likelihood ratio. The cut-off value for the calculated specificity and sensitivity was <0.201 pmol/ml.

### Reduced cGMP levels in AD patients with current depression

**Fig. 4:**
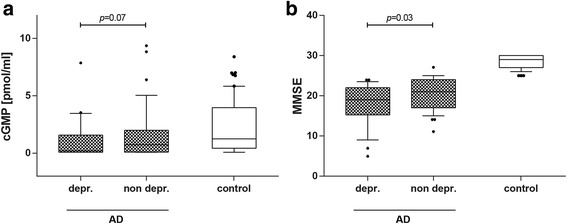
AD patients with current depression show a decrease in CSF levels of cGMP and significantly lower MMSE scores. **a** CSF concentration of cGMP is decreased in AD patients suffering from current depression compared with non-depressed AD patients (*p* = 0.07, Mann–Whitney *U* test). **b** AD patients with current depression show significantly lower MMSE scores (*p* = 0.03, Mann–Whitney *U* test). *AD* Alzheimer’s disease, *cGMP* cyclic guanosine-3',5'-monophosphate, *depr.* depression, *MMSE* mini-mental state examination

To test the question of whether depression as the most common early sign of NPSs affects cGMP levels in AD patients, we subgrouped the AD patients by diagnosis of current depression and compared them with non-depressed AD patients. To exclude that the intake of antidepressants alone confounds the measurements of cyclic nucleotide levels, we first tested whether subjects receiving antidepressants show altered cAMP or cGMP levels compared with non-recipients. We did not see a statistically significant difference (Additional file 1: Figure S1). In contrast, we observed that CSF cGMP levels are reduced (~73%, *p* = 0.07) in AD patients suffering from current depression compared with non-depressed AD patients (Fig. 4a). The median of concentrations was 0.20 (0.08–1.6) pmol/ml (current depression) and 0.73 (0.10–2.00) pmol/ml (non-depression).

Our analyses further revealed that there is a statistically significant reduction in MMSE scores for AD patients suffering from current depression compared with non-depressed AD patients (~10%; *p* = 0.03) (Fig. 4b).

**Fig. 5:**
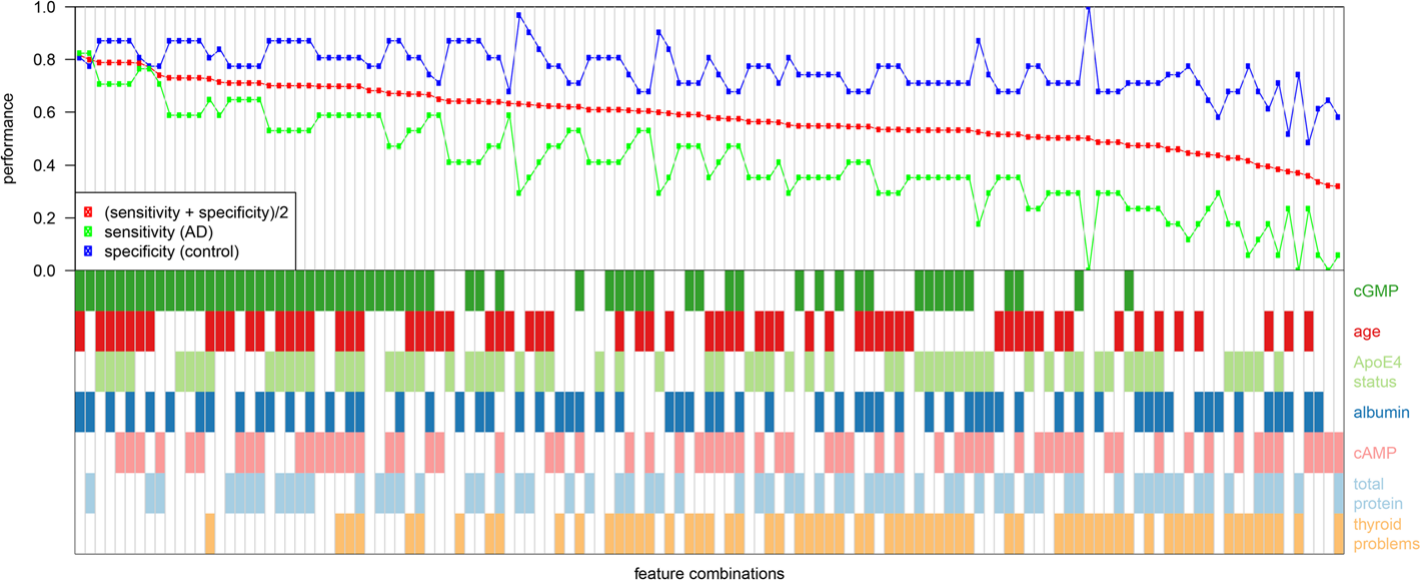
Exhaustive evaluation of marker combinations in a depressive subgroup. *Top*: cross-validation performances of all tested parameter combinations: *left*, models with higher mean of sensitivity and specificity; *right*, models with lower performance. *Bottom*: corresponding parameter combinations: *coloured rectangle*, corresponding parameter is used in the model; *white rectangle*, corresponding parameter is not used. The different parameters are ranked according to their frequency in the top models. In the top-performing models, “cGMP” is used most frequently followed by “Albumin” and “AgeAtLP”. *AD* Alzheimer’s disease, *cAMP* cyclic adenosine-3',5'-monophosphate, *cGMP* cyclic guanosine-3',5'-monophosphate (Colour figure online)

For the subgroup of samples with depression, all combinations of the biomarkers total protein, albumin, ApoE4 status, cGMP, cAMP, age and thyroid problems were tested for their predictive performance (AD vs control). Fig. 5 shows that accuracies of up to 81.5% were reached (biomarker combination: “cGMP”, “AgeAtLP”, “Albumin”). By looking at all combinations it can be seen that cGMP is an important component because it is part of all top-performing models with an accuracy > 70% (19 models).

## Discussion

The loss of synapses in memory-related brain areas is a very early event in the disease process of AD and the strongest structural correlate with the decrease in cognitive performance of patients [31, 32]. Synaptic plasticity is a crucial characteristic of neurons that is thought to underlie memory and learning [33–35]. One important regulator of synaptic plasticity is the signalling of cyclic nucleotides [36, 37], which is in turn impaired in the CNS of AD patients [38–42]. CREB, the common downstream target of both cAMP and cGMP signalling and regulator of structural correlates of cognition, is also affected in AD brains [6, 43, 44]. Because of the fact that pathological processes of AD start before the onset of clinical manifestations [45–47], the identification of novel biomarkers is of crucial interest. Up to now only two studies have been performed analysing the levels of the cyclic nucleotides cAMP and cGMP in the CSF of patients suffering from AD [14, 15]. The inconsistency in results may be due to limited cohort sizes (*n* = 20 [15]; *n* = 79 [14]) and different study designs. Thus, in the present study we investigated the concentrations of the cyclic nucleotides cAMP and cGMP in the CSF of a well-characterized larger cohort of AD patients and compared them with non-AD controls.

As a main activator of CREB, a decrease in cAMP signalling has been reported in the hippocampus and temporal cortex of AD patients [48, 49]. In these studies cAMP levels were not determined directly. Yamamoto et al. [48] determined the hippocampal levels of the neural specific adenylyl cyclase type I (cAMP synthesis) and found these to be decreased in AD cases. Another study conducted by Kim et al. [49] revealed that PKA (specific downstream target of cAMP) levels are lower in AD cortices. Regarding PDE expression in AD brains, the severest clinical stages are associated with an increase in PDE8B expression in memory-associated brain regions, such as the entorhinal cortex [50]. Additionally, chronic inhibition of PDE4D (cAMP specific) led to improved spatial memory function in APPswe/PS1dE9 mice [51]. Demonstrating altered levels of cAMP synthesizing/degrading enzymes in AD brains, these studies provide further evidence for a reduction in cAMP signalling in affected patients. Furthermore, it has been shown that a deregulation in cAMP signalling plays a role in the pathology of other neurodegenerative diseases such as major depressive disorder (MDD) [52], multiple sclerosis (MS) [53] and striatal motor disorders like Parkinson’s disease (PD) and Huntington’s disease (HD) [54]. With regard to the use of cAMP as a biomarker of neurodegenerative disease, and AD in particular, different studies were conducted with contradictory findings. For PD, some groups found a decrease in cAMP levels [55, 56] and others showed no statistically significant difference [57, 58]. Oeckl et al. [57] reported a significant decrease of cAMP levels in the CSF of Creutzfeldt-Jakob disease (CJD) patients compared with controls. Two studies specifically investigated the CSF levels of cAMP of AD patients. Martínez et al. [15] found elevated cAMP CSF levels in patients suffering from AD compared with non-demented controls. In contrast to these results, a recently published study reported no changes in cAMP levels in the CSF of AD patients compared with non-demented controls [14]. In line with the second study, we did not find statistically significant changes in cAMP levels in the CSF of AD patients compared with our control cohort. These results confirm that cAMP may not be used as a marker of disease progression and severity in AD patients.

Similarly to cAMP, the cyclic nucleotide cGMP is also strongly linked to memory processes and is thought to be altered in AD pathology [41]. However, compared with cAMP, fewer studies determined changes in cGMP signalling in AD patients. To the best of our knowledge there are no published data determining cGMP levels in the human brain directly. This could be due to stability problems of cyclic nucleotides in post-mortem tissue. Ugarte et al. [14] found increased PDE5A (cGMP degrading) expression in AD brains in a cohort of eight controls and seven AD cases, potentially indicating decreased cGMP in these AD brains. Furthermore, Reyes-Irisarri et al. [59] determined the expression of cGMP-specific PDE2 and PDE9 in control and AD brains. Their study showed no PDE5 mRNA expression in the human brain and no changes in PDE2 or PDE9 expression, when comparing AD patients and controls. The differences in results of both studies could be explained by using different methods (real-time PCR vs in-situ hybridization) or too small a number of brain samples to conclude physiological consequences for AD pathology. Regarding transgenic AD rodent models, Jin et al. [60] were able to show that treatment with a PDE5 inhibitor resulted in increased cGMP levels in the cortex and hippocampus and improved learning and memory functions in APP/PS1 transgenic mice. Furthermore, Cuadrado-Tejedor et al. [61] demonstrated that the PDE5 inhibitor sildenafil reversed cognitive deficits in Tg2576 mice compared with age-matched litter mates. Using APP/PS1 transgenic mice, Puzzo et al. [62] showed that PDE5 inhibition improves synaptic function and memory in these mice. All three of these studies support an important role of cGMP in AD pathophysiology with or without modification of brain amyloid burden. More striking evidence for a crucial contribution of cGMP signalling is provided by studies analysing the effects of PDE9 inhibition in AD transgenic mice. For example, Kroker et al. [9] demonstrated recently that a PDE9A inhibitor is capable of restoring Aβ_42_ oligomer-impaired LTP in rat hippocampal slices. They further showed that PDE9A inhibitor treatment of Tg2576 mice enhanced cGMP levels in the hippocampus and resulted in improved memory performance of these mice. PDE2 can hydrolyse both cAMP and cGMP. Sierksma et al. [63] showed that inhibition of PDE2 improves impaired memory in the APPswe/PS1dE9 mouse model. Nevertheless, further studies are needed to answer the question of whether potentially altered PDE expression in brains of AD patients is causative for or a consequence of the disease.

Most of the aforementioned studies determining CSF cAMP levels also analysed the levels of cGMP in the CSF of patients suffering from a neurodegenerative disease compared with controls. These findings were also conflicting. Oeckl et al. [57] showed a significant decrease in the CSF cGMP levels of CJD patients, but not PD or ALS patients. In line with these findings, two other studies did not find any differences in the CSF cGMP levels of PD patients [56] or PD and ALS patients [64]. In contrast to this, Belmaker et al. [55] found a significant decrease of cGMP in PD patients. However, dementia was not determined in any of the PD studies. Martínez et al. [15] and Ugarte et al. [14] had been the only groups to analyse cGMP levels in the CSF of AD patients until now. While the former study did not show statistically significant differences in the cGMP levels of AD patients, Ugarte et al. report a decrease of cGMP levels in mild AD patients (MMSE 22.3 ± 0.7) compared with different controls. We found in our study a reduction of about 40% in cGMP levels in the CSF of AD patients compared with the control group. Our results revealed a significant correlation of CSF cGMP levels with the cognitive performance (MMSE score) of all patients. Therefore we stratified the AD patients by their MMSE scores into three clusters (severe AD, moderate AD, mild AD). This showed that the observed decrease in AD patients is mainly driven by more severely affected patients with an MMSE score of between 5 and 17 points. Because disturbed intracellular mechanisms are thought to occur decades before the appearance of clinical symptoms, cGMP levels may at least not be an early indicator for intracellular changes in AD brains. Intracellular cGMP levels are tightly regulated and kept on a physiological concentration by three mechanisms, namely synthesis via guanylyl cyclases, degradation via PDEs and excretion by transport proteins. Therefore, altered pathological levels may not be observed before synapses and neurons are degenerating. Intracellular compensating mechanisms (GCs, PDEs) may counterbalance subtle changes in cGMP levels in preclinical and prodromal AD. Taken together, these results provide further evidence for an involvement of cGMP/NO-dependent pathways in AD pathology. The ELISA kits used by Martínez et al. [15] to determine cAMP and cGMP levels in CSF differed from ours. However, the main drawback of their study is the use of headache patients as a control group and the lack of neuropsychological tests for control subjects. These facts might, at least in part, explain the contradictory findings. Nevertheless, this should be taken into consideration in future study designs, because headache patients are commonly used as controls in neurological examinations and the involvement of cGMP/NO-dependent pathways in cephalalgia onset is discussed in the neurological community [65–68].

The fact that only cGMP and not cAMP levels are decreased in the CSF of AD patients in our study might be explained by an increased activity of cGMP-specific PDEs in the CNS of AD patients [41]. Another explanation could be a reduction of guanylyl cyclase activity in AD brains [11, 69]. Given that there is a massive neuron loss in AD brains and we report a negative correlation of CSF cGMP with CSF tau, a reduction in guanylyl cyclase activity seems to be more likely than increased activity of PDEs. An alteration in cGMP efflux to the extracellular space by specific transporters might also be a possibility [70]. Potential age-dependent differences in CSF cyclic nucleotide levels might not be a relevant explanation, because we and others showed that levels of cyclic nucleotides do not alter with aging (data not shown) [71]. Different measurement values due to freezing/thawing cycles can also be ruled out, because no stability problems with cyclic nucleotides were observed [57]. Moreover, levels of cyclic nucleotides were reported not to be subject to changes in circadian rhythm [71].

Deeper understanding of the underlying pathophysiology of AD and accompanying NPSs is crucial, because symptoms such as late-life depression are highly prevalent and lead to poor medical and functional outcomes. Because late-life depression can be both a risk factor for and consequence of AD, we subgrouped the AD cohort into AD cases with current depression and AD patients without current depression. We found that cGMP levels are reduced in AD patients suffering from current depression compared with non-depressed AD patients, indicating a relationship between the glutamatergic system and NPSs. We were also able to demonstrate significantly lower MMSE scores in depressed AD patients. The glutamatergic system is the molecular correlate of cognitive function, and lower MMSE scores in AD patients suffering from late-life depression are also in line with an involvement of the glutamatergic system in depressive syndromes. Increasing evidence supports the involvement of the glutamatergic system, including neuroplasticity, in the pathophysiology of MDD. In mice, stress can reduce dendritic complexity in the CA3 region of the hippocampus, which could be reverted by serotonin reuptake inhibitors [72]. Further, structural imaging revealed hippocampal atrophy in MDD patients [73, 74]. An increase of cholinergic activity is also described to be involved in pathophysiology of MDD [75]. Interestingly, cGMP is discussed to be a second messenger in acetylcholine signalling [76]. Given that cholinergic signalling is discussed to be enhanced in depression but decreased in AD brains [77, 78], impaired acetylcholine action as a common mechanism explaining our findings seems to be unlikely. NPSs could either be a psychological reaction to experienced cognitive decline or a risk factor for AD by affecting the brain (e.g. by activating the neuroendocrine axis) [79]. Whether the reduction in cGMP levels is causative or a consequence of NPSs, such as late-life depression, is still not known. Understanding these symptoms in more detail is crucial, because their phenotype can help to characterize the involved circuitries and neural regions, and thereby offer clues about AD pathogenesis.

cGMP levels depicted only moderate discrimination power in ROC analyses in our cohort. This may indicate that cGMP alone is not a useful biomarker in clinical routine, but can help in understanding the pathophysiological background of AD and other neurodegenerative diseases related to synaptic plasticity deficits. The power of prediction could be increased when analysing cGMP levels in CSF together with other markers. Nevertheless, levels of cGMP in CSF might serve as a valuable marker for monitoring target engagement of PDE inhibitors in clinical trials, disease progression or possibly therapeutic efficacy, because such inhibitors are considered to have high potential as future treatment options against cognitive decline in AD patients [80, 81]. However, their therapeutic potential for AD patients with or without NPSs still needs to be shown in clinical trials. Nonetheless, as regards the measurement of cGMP in CSF, it is of great importance to improve standardization in the study design, inclusion criteria, choice of suitable control group, sample storage and handling, and choice of assays. The strengths of the current study are the sample size and the use of both clinical and CSF biomarker data as diagnostic criteria, as well as the exclusion of headache co-morbidity as a potential confounder and the detailed clinical characterization of the AD group. One advantage of commercial ELISA kits compared with other methods such as LC-MS/MS is their potential for standardization and their usability in clinical routine among different laboratories. Because we were able to show that only severely demented AD patients and AD patients suffering from current depression exhibit decreased CSF cGMP levels, one has to be aware of the degree of cognitive impairment and potential co-morbidities, such as depressive syndromes, of patients when assembling cohorts. The inclusion criteria should be considered carefully, making the obtained data more reliable and comparable.

## Conclusions

The present study provides further evidence for a specific involvement of cGMP in AD pathogenesis and accompanying co-morbidities, such as NPSs. We found that cGMP shows moderate discrimination for clinical routine. Our data provide additional support from human material for deciphering the pathophysiological background of AD related to synaptic dysfunction. Understanding associated neuropsychological symptoms in the etiopathology of neurodegenerative diseases in more detail is essential, because their phenotype might help to delineate the involved neural regions and circuitries and thereby offer clues about pathogenesis of neurodegenerative diseases.

## Additional file

Additional file 1: Figure S1. is showing cGMP and cAMP levels in CSF of subjects taking antidepressants compared with subjects not taking antidepressants separated into control and AD groups. **A** CSF cGMP levels were not altered in control subjects taking antidepressants compared with controls who did not take antidepressants (*p* = 0.79). **B** CSF cAMP levels were not altered in control subjects taking antidepressants compared with controls who did not take antidepressants (*p* = 0.51). **C** CSF cGMP levels were not altered in AD patients taking antidepressants compared with AD patients who did not take antidepressants (*p* = 0.60). **D** CSF cAMP levels were not altered in AD patients taking antidepressants compared with AD patients who did not take antidepressants (*p* = 0.38). *Dark horizontal lines*, mean of observed data; *box*, 25th and 75th percentiles; *whiskers*, 5th and 95th percentiles; *dots*, outliers. *p* values calculated using the Mann–Whitney rank-sum test. (TIF 514 kb)

## Abbreviations

AD: Alzheimer’s disease
ALS: Amyotrophic lateral sclerosis
Aβ: Beta-amyloid
BDI: Beck Depression Inventory
cAMP: Cyclic adenosine-3′,5′-monophosphate
cGMP: Cyclic guanosine-3′,5′-monophosphate
CREB: cAMP-response element binding protein
CSF: Cerebrospinal fluid
MDD: Major depressive disorder
MMSE: Mini-Mental State Examination
NO: Nitric oxide
NPS: Neuropsychiatric symptom
PDE: Phosphodiesterase
