# Supplementary figures and images for: Reduced cGMP levels in CSF of AD patients correlate with severity of dementia and current depression
Source: Alzheimers Res Ther. 2017 Mar 9;9:17. doi: 10.1186/s13195-017-0245-y (PMC5343324; doi:10.1186/s13195-017-0245-y)

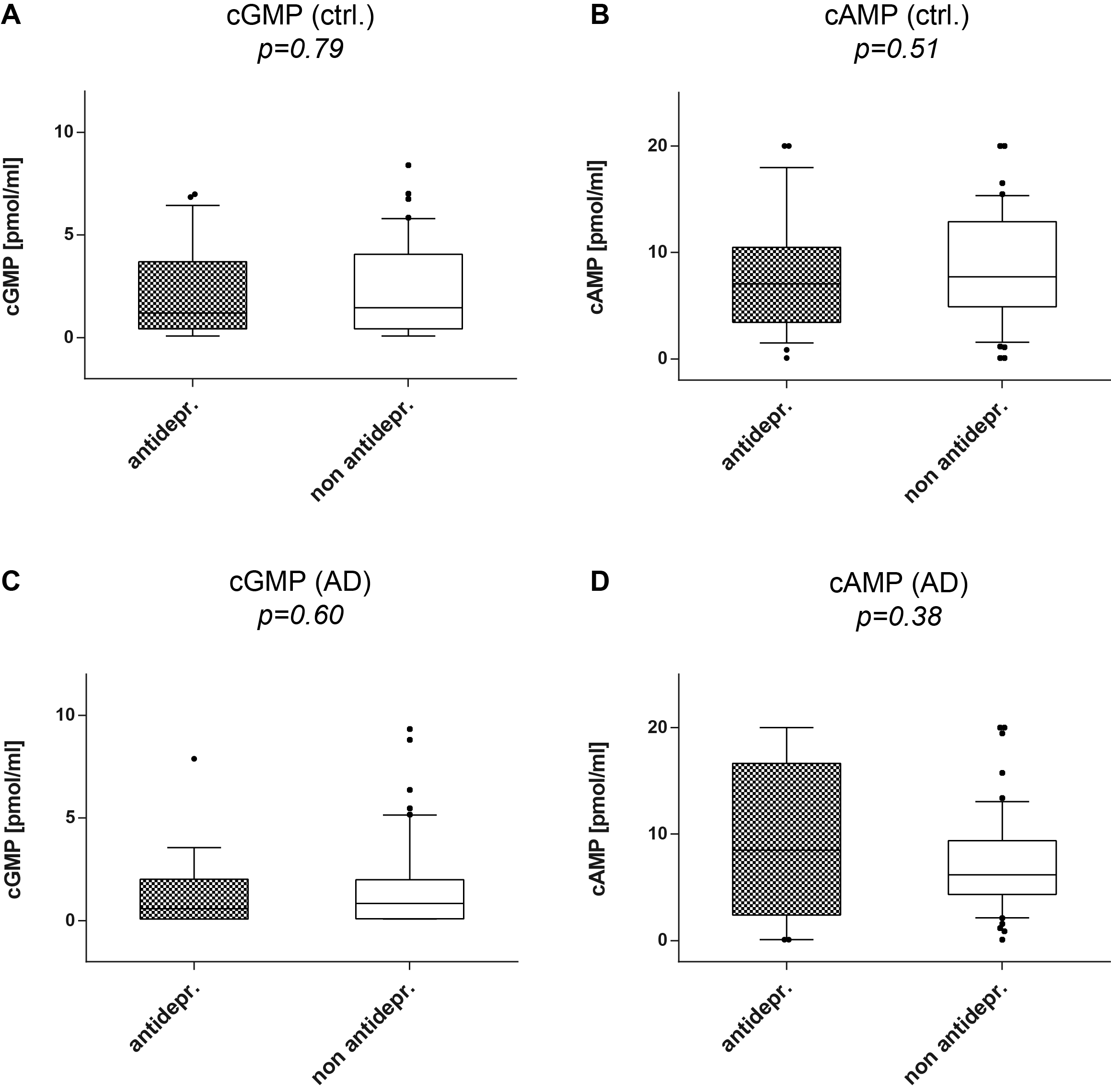

Supplement: Additional file 1: Figure S1. — is showing cGMP and cAMP levels in CSF of subjects taking antidepressants compared with subjects not taking antidepressants separated into control and AD groups. A CSF cGMP levels were not altered in control subjects taking antidepressants compared with controls who did not take antidepressants (p = 0.79). B CSF cAMP levels were not altered in control subjects taking antidepressants compared with controls who did not take antidepressants (p = 0.51). C CSF cGMP levels were not altered in AD patients taking antidepressants compared with AD patients who did not take antidepressants (p = 0.60). D CSF cAMP levels were not altered in AD patients taking antidepressants compared with AD patients who did not take antidepressants (p = 0.38). Dark horizontal lines, mean of observed data; box, 25th and 75th percentiles; whiskers, 5th and 95th percentiles; dots, outliers. p values calculated using the Mann–Whitney rank-sum test. (TIF 514 kb) [file 13195_2017_245_MOESM1_ESM.tif]
